# Oral Administration of Efavirenz Dysregulates the *Tph2* Gene in Brain Serotonergic Areas and Alters Weight and Mood in Mice

**DOI:** 10.3390/ph17060801

**Published:** 2024-06-18

**Authors:** Sandra Angélica Rojas-Osornio, Minerva Crespo-Ramírez, Vladimir Paredes-Cervantes, Antonio Mata-Marín, Ricardo Martínez-Lara, Miguel Pérez de la Mora, Emiliano Tesoro-Cruz

**Affiliations:** 1 Escuela Superior de Medicina, Instituto Politécnico Nacional, Mexico City 11340, Mexico; sandii38@yahoo.com.mx; 2 Division de Neurociencias, Instituto de Fisiología Celular, Universidad Nacional Autónoma de México, Mexico City 04510, Mexico; mcrespo@ifc.unam.mx; 3 Laboratorio Central, Hospital de Especialidades “Dr. Antonio Fraga Mouret” Centro Médico Nacional “La Raza” Instituto Mexicano del Seguro Social, Mexico City 02990, Mexico; vlapace@hotmail.com; 4 Departamento de Infectología, Hospital de Infectología del Centro Médico Nacional “La Raza” IMSS, Mexico City 02990, Mexico; jamatamarin@gmail.com; 5 Unidad de Investigación Biomédica en Inmunología e Infectología, del Hospital de Infectología del Centro Médico Nacional “La Raza” IMSS, Mexico City 02990, Mexico; rml900_z@hotmail.com

**Keywords:** efavirenz, depression, tryptophan hydroxylase 2, 5-HT, anxiety

## Abstract

Most HIV-antiretroviral drugs have adverse effects. Efavirenz (EFV) is an example of a drug with neuropsychiatric effects, such as anxiety, depression, and suicidal thoughts, in people living with HIV (PLWH). The mechanisms by which EFV causes neuropsychiatric alterations in PLWH are complex, multifactorial, and not fully understood, although several studies in animals have reported changes in brain energy metabolism, alterations in monoamine turnover, GABA, and glutamate levels, and changes in 5-HT receptors. In this report, we studied the effects of EFV on the serotonergic system in healthy mice, specifically, whether EFV results in alterations in the levels of the tryptophan hydroxylase 2 (*Tph2*) gene in the brain. EFV (10 mg/kg) and distilled water (1.5 µL/kg) (control group) were orally administered to the mice for 36 days. At the end of the treatment, Tph2 expression levels in mouse brains were measured, and mood was evaluated by three trials: the forced swim test, elevated plus maze, and open field test. Our results revealed dysregulation of Tph2 expression in the brainstem, amygdala, and hypothalamus in the EFV group, and 5-HT levels increased in the amygdala in the EFV group. In the behavioral tests, mice given EFV exhibited a passive avoidance response in the forced swim test and anxiety-like behavior in the elevated plus maze, and they lost weight. Herein, for the first time, we showed that EFV triggered dysregulation of the *Tph2* gene in the three serotonergic areas studied; and 5-HT levels increased in the amygdala using the ELISA method. However, further studies will be necessary to clarify the increase of 5-HT in the amygdala as well as understand the paradoxical decrease in body weight with the simultaneous increase in food consumption. It will also be necessary to measure 5-HT by other techniques different from ELISA, such as HPLC.

## 1. Introduction

Efavirenz, a nonnucleoside reverse transcriptase inhibitor (NNRTI), has been a first-line component of antiretroviral therapy worldwide for several years. However, it has been reported to trigger abnormal dreams, sleep disturbances, nervousness, anxiety, depression, and dizziness in as many as 25–40% of treated people living with HIV (PLWH) [1,2,3]. Although some side effects gradually disappear within one month, most patients experience persistent side effects in the central nervous system [4,5,6], and this could be explained by the CYP2B6 polymorphisms that some populations have [7,8,9,10,11,12], which provide variations in the general pharmacokinetics that trigger a high plasma concentration or longer plasma half-life of efavirenz [7,9,11,13,14,15,16,17,18].

In addition, animal studies in rodents have reported changes in brain energy metabolism, especially in the cerebral cortex, striatum, and hippocampus [19,20]. Alterations in monoamine turnover [21], GABA, and glutamate levels were also reported in the rat striatum [22].

Streck E.L. et al., 2008 showed that most enzymatic effects appeared after oral administration of EFV: creatine kinase inhibition activity in cerebellum, hippocampus, striatum, and cortex when 10 mg/kg was given once a day for 36 days to mice [23]. Moreover, Edagha I.A. et al., 2022 showed that antioxidants (superoxide dismutase, catalase, and glutathione peroxidase) decreased after administration of EFV/Lamivudine/Tenofovir disproxil fumerate at a dose 17.14 mg/kg for 30 days to rats [24]. Anxiety, depression, and cognitive performance were also found in rodents after chronic (30, 34, or 36 days) oral administration of the drug in a 10 mg/kg dose [25,26,27].

Ntshangase S. et al., 2019, showed a high degree of EFV localization across the entire brain by matrix-assisted laser desorption ionization mass spectrometry imaging (MALDI-MSI) in rats after oral administration of the drug (50 mg/kg) [27]. Moreover, EFV is widespread throughout the brain, highly distributed in the cerebral cortex, corpus callosum, basal forebrain, globus pallidus, and hippocampal formation, 0.5 h post-dose administration [27].

Several studies have shown that 5-HT signaling plays a critical role in anxiety-related behaviors, depression development, and suicidal thoughts [28]. Moreover, the involvement of 5-HT at the heart in these effects is rather appealing. Since tryptophan hydroxylase 2 (Tph2) is the rate-limiting step for brain 5-HT biosynthesis [29,30], it was recently shown to enhance Tph2 activity within the raphe nucleus after in vivo transfection in mice following the ocular instillation of a plasmid (pIRES-hrGFP-1a-Tph2-FLAG) [30].

Therefore, this work aimed to study whether EFV oral administration results in alterations in Tph2 expression by qPCR and 5-HT levels by ELISA in selected serotonergic system brain regions, such as the brainstem, hypothalamus, and amygdala, and whether these changes are associated with the development of anxiety and/or depressive-like behavior in mice via behavioral tests. However, it is important to highlight that the 5-HT level is more sensitive to measurement by HPLC than ELISA.

## 2. Results

### 2.1. Effects of EFV on Tph2 Expression in the Brainstem, Hypothalamus, and Amygdala in Mice

**Figure 1:**
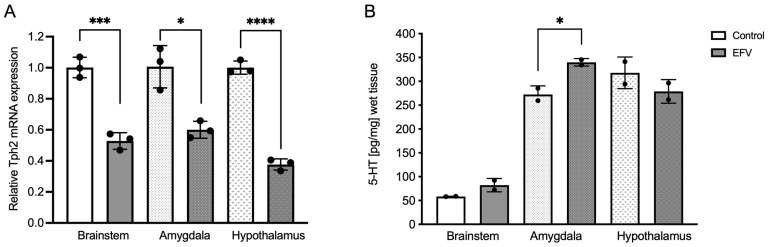
Effects of EFV on Tph2 expression (**A**) and 5-HT levels (**B**) in the brainstem, amygdala, and hypothalamus in mice. Diminished Tph2 mRNA expression following EFV administration compared to that in the control group is shown (**A**). Each dot represents three pooled samples of tissues from three different animals. In contrast, 5-HT levels were increased after EFV administration only within the amygdala (**B**). Each dot represents two pooled samples of tissues from three different animals. Unpaired *t* test with Welch’s correction. * *p* < 0.05; *** *p* < 0.001; **** *p* < 0.0001.

To determine whether EFV treatment had any effect on brain Tph2 mRNA expression within the brainstem, hypothalamus, or amygdala, qPCR was performed after 36 days of antiretroviral treatment. Unpaired *t* tests with Welch’s correction analysis revealed significant differences in the EFV within the brainstem (F_2,2_ = 1.519; *p* < 0.001), amygdala (F_2,2_ = 6.208; *p* < 0.05), and hypothalamus (F_2,2_ = 1.396, *p* < 0.0001) (Figure 1A).

### 2.2. Effects of EFV on 5-HT Levels in the Brainstem, Hypothalamus, and Amygdala in Mice

To gain insight into the likely functional correlation between Tph2 expression and 5-HT synthesis, the levels of this neurotransmitter were also measured in the regions in which Tph2 was expressed. Unlike Tph2 expression, an unpaired *t* test with Welch’s correction revealed a statistically significant increase in the 5-HT level within the amygdala following EFV treatment (F _2,2_ = 4.819, *p* < 0.05) (Figure 1B).

### 2.3. Effects of EFV on Behavior in Mice

**Figure 2:**
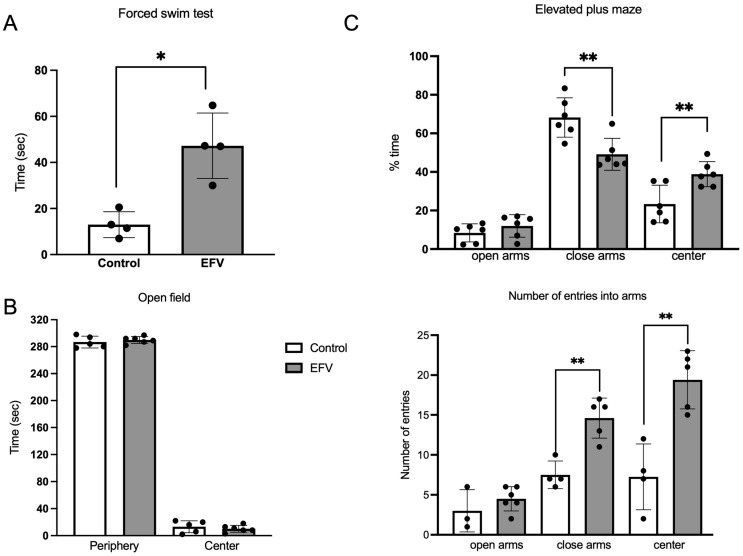
Behavioral tests. Three behavioral paradigms were used in this study to assess depression-like behavior (force swim test) or anxiety (open field test and elevated plus-maze test) in mice following 36 days of oral EFV administration (10 mg/kg). (**A**) A significant increase in immobility time was observed in the EFV group compared to the control group. Unpaired *t* test with Welch’s correction (F_3,3_ = 6.409; *p* < 0.05). (**B**) In the open field test, no difference was detected between the EFV group and the control group (Welch’s *t* test; F_4,5_ = 2.950; *p* > 0.05). (**C**) In the elevated plus maze test, a significantly lower percentage of time spent in close arms and in the center was observed in mice treated with EFV, and the percentage of entries increased (Welch’s *t* test; *p* < 0.01). The percentage of time spent on the EPM test was calculated as the time spent on the arms in seconds divided by the total duration spent on the EPM test, which was 300 s × 100%. Each dot represents an animal. The values are the means ± SEMs. Welch’s *t* test. * *p* < 0.05; ** *p* < 0.01.

Unpaired *t* tests with Welch’s correction revealed a significant increase in immobility time in the EFV-treated group in the forced swim test (FST) (F_3,3_ = 6.409; *p* < 0.05) (Figure 2A). No differences were detected between groups in the open field test (OFT) (F_4,5_ = 2.950; *p* > 0.05) (Figure 2B). Although no difference was observed in the time spent in the open arms (F _5,5_ = 1.542; *p* > 0.05) in the elevated plus maze (EPM), mice treated with EFV spent less time in the closed arms (F _5,5_ = 1.528; *p* < 0.01) or in the center (F _5,5_ = 2.257; *p* < 0.01) than did the control (Figure 2C). Furthermore, the number of entries to the closed arms (F _5,5_ = 2.100; *p* < 0.01) and center (F _5,5_ = 1.272; *p* < 0.01) was greater than that in the control group (Figure 2C).

### 2.4. Effects of EFV on Body Weight and Food Intake in Mice

**Figure 3:**
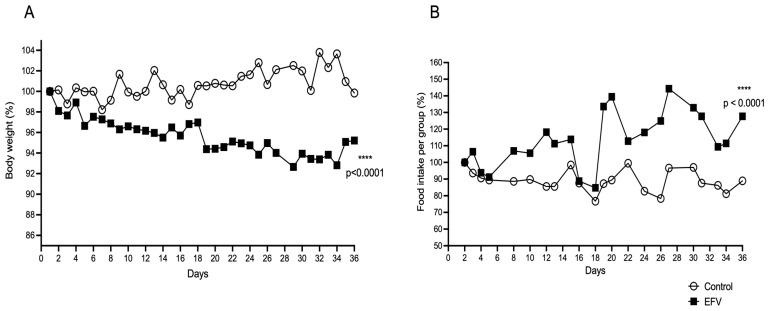
Effects of EFV on body weight (**A**) and food consumption (**B**). Twenty-four mice per group were fed and housed in groups of six with water available ad libitum in each home cage (*n* = 48, 8 cages). Every day, the mice received EFV (10 mg/kg) via the oral route or distilled water (1.5 µL/kg) via the oral route to determine whether EFV alters body weight or feeding. The body weight of each mouse and the food consumption of each cage were measured daily. This experiment continued until 36 days after EFV administration. A significant decrease in body weight was observed in the EFV group, beginning with respect to the final weight (F_35,74_ = 4.828; *p* < 0.0001). With respect to food intake, an increase in consumption was observed in the EFV group compared with the control group (F_21, 21_ = 6.498; *p* < 0.0001). The distribution of the values of the studied parameters was tested for normality using the Shapiro–Wilk test. Body weight was analyzed with two-way repeated-measures ANOVA followed by the Tukey post hoc test. Food intake was analyzed by an unpaired *t* test. The values are the means ± SEMs. **** *p* < 0.0001.

Chronic administration of EFV resulted in a significant decrease in the body weight of the mice compared to the initial weight (one-way ANOVA, post hoc Tukey test, F_35,74_ = 4.828; *p* < 0.0001), unlike that of the control mice (one-way ANOVA, post hoc Tukey test, F_35,73_ = 0.166; *p* > 0.05) (Figure 3A). Regarding food intake (Figure 1B), mice treated with EFV showed an increase in food intake. Unpaired *t* test (F_21,21_ = 6.498; *p* < 0.0001).

## 3. Discussion

Thus far, antiretroviral drugs have made our fight against AIDS possible, increasing the life expectancy of people suffering from this disease. EFV is a potent, safe, and tolerable nonnucleoside reverse transcriptase inhibitor (NNRTI) recommended as an initial therapy and in combination with other antiretrovirals [19,31]. However, its use has decreased because adverse neuropsychiatric side effects have been reported in patients [31,32,33], and its clinical use has been limited in many countries.

Although there is a paucity of information on the effects of EFV on neural function, several reports, both in humans and rodents, have contributed to the understanding of brain function and neuropsychiatric side effects [6,20,34]. Therefore, our results have directed part of this understanding, providing new information regarding the alteration of the serotonergic system and revealing that EFV triggered dysregulation of the Tph2 gene in the three serotonergic areas studied: the brainstem, hypothalamus, and amygdala. However, we expected a decrease in 5-HT levels due to dysregulation of the Tph2 gene (Figure 1), but this effect was not detected.

Nevertheless, using the ELISA method to measure 5-HT, the levels increased in the amygdala of mice treated with EFV. Nonetheless, the increase in 5-HT levels in the amygdala could be explained by the fact that EFV has an inhibitory effect on monoaminoxidase (MAO), which was well established and explained by Zareifopoulos N. et al., 2020 [35]. However, it is important to mention that indolalkylamines (including 5-HT) as catecholamines are quickly oxidized at neutral PH. 7.5. Therefore, it will be necessary to measure the levels of serotonin with another technique more selective, such as high-pressure liquid chromatography (HPLC).

Moreover, we make it clear the fundamental limitations of the current work, which means that it was not possible to demonstrate by Western blot and mass spectroscopy the protein levels of TPH2, SERT, and MAO. Hence, there will be interest in future works to measure the protein expression level of several important markers like SERT, MAO, and different 5-HT receptors.

On the other hand, Frick et al., 2015, examined patients with social anxiety disorders (SAD) and reduced serotonin transporter availability using positron emission tomography (PET) and found increased 5-HT synthesis in the amygdala, raphe nuclei region, caudate nucleus, putamen, hippocampus, and anterior cingulate cortex of patients characterized by an overactive presynaptic serotonin system [36]. In another report by Hanley et al., 2002, a decrease in the expression of the Tph2 gene caused a decrease in the amount of available 5-HT; however, they also observed that short exposure of the 5-HT(2A) receptor to its ligand causes desensitization, which has been associated with attention deficit and hyperactivity disorder [37]. In contrast, Waider J. et al., 2013 reported that a life-long reduction or complete lack of brain 5-HT transmission causes differential changes in GABA systems in limbic regions (especially in the amygdala), which are key players in emotional learning and memory processes. These changes likely reflect a combination of developmental alterations and functional adaptations of emotion circuits to balance the lack of 5-HT and may underlie altered emotional behavior in 5-HT-deficient mice [38]. Our findings of mood alteration caused by an imbalance of 5-HT were corroborated by behavioral assays in mice after 36 days of EFV oral administration, where depression-like behavior and anxiety were observed in the FST and EPM tests, respectively (Figure 2), which corroborates results from other reports [25,39].

Additionally, as a limitation of this work, we used six animals in each behavioral group. We used the minimum possible number of animals to obtain statistically reliable results. However, a larger number, perhaps twice as many animals, allows for data with less error is even more reliable.

In the open field test (OFT), mice treated with EFV showed a passive avoidance response, and no differences were observed between the groups. However, other reports have shown that social avoidance and avoidance of bright spaces are linked to amygdala neurons via 5-HT_1_ receptors (HTR_1A_ and HTR_1B_). However, pharmacological inhibition of HTR_1A_ and HTR_1B_ in the basal amygdala induces avoidance of bright spaces and social avoidance, respectively. Inactivation or desensitization of these receptors can cause depressive or anxiogenic effects [40,41]. Dalwadi et al., 2016 described the interactions of different cloned metabotropic 5-HT receptors, including the 5-HT_6_ receptor and the 5-HT_2_ subfamily of receptors (5-HT_2A_, 5-HT_2B_, and 5HT_2C_), with EFV, but no significant interactions were detected with the 5-HT_1_ receptor [6]. However, an indirect effect on the 5-HT_1_ receptor could be explained by increasing or decreasing 5-HT levels as a consequence of EFV via the Tph2 gene alteration observed in the present work, but further studies will be necessary to understand the interaction between the 5-HT_1_ receptor and EFV.

Instead, Gatch M.B. et al., 2013 showed that EFV has also been associated with effects such as lysergic acid diethylamide (LSD), which is a psychoactive drug that is mediated by the 5-HT_2A_ receptor in a dose-dependent manner, causing adverse neuropsychiatric events because it is a partial agonist of the 5-HT_2A_ receptor [42] and acts as a serotonin 5-HT_2A_ receptor antagonist, a serotonin-dopamine reuptake inhibitor, an inhibitor of monoamine oxidase A (MAO), a vesicular monoamine transporter 2 (VMAT2) inhibitor, a 5-HT_2B_ antagonist, and a 5-HT_6_ inverse agonist [37]. These interactions of EFV with serotonergic targets, such as the prefrontal cortex, thalamus, cerebellum, amygdala, hippocampus, and hypothalamus, have been associated with neuropsychiatric adverse effects (NPAEs) and are related to alterations in serotonin levels.

In the present study, for the first time, we showed that the chronic administration of EFV to healthy mice led to Tph2 dysregulation within the brainstem, hypothalamus, and amygdala, which triggered an increase in 5-HT levels only in the amygdala. However, further studies will be necessary to clarify the increase in 5-HT levels. Moreover, considerable evidence suggests that disturbances in 5-HT neuronal activity occur at the heart of depression [43,44], and the tryptophan hydroxylase-2 gene (Tph2), which encodes Tph2, the rate-limiting enzyme of 5-HT synthesis in the brain, modulates the responses of limbic circuits to adverse emotional stimuli [45]. Thus, it is conceivable that the reduced Tph2 gene expression found in this work triggered a decrease in Tph2 activity, resulting in a decrease in extracellular 5-HT levels and a probable reduction in 5-HT autoreceptor signaling, leading to an increase in serotonin levels in the tissue, which is another factor that contributes to the explanation of the NPAEs caused by EFV.

Finally, in this work, we observed involuntary weight loss in mice that received EFV for 36 days since their food intake did not decrease; on the contrary, it was greater compared to the control group (Figure 3). Both parameters could be related to the probable agonist effect of EFV on the function of central nervous system-specific 5-HT_2C_ receptors, which are distributed in the hypothalamus, substantia nigra, and choroid plexus. Reports have established that those receptors control key physiological functions, such as food intake, anxiety, and motoneuron activity that regulates corticotropin release in response to stress, involving hypothalamic neurons that produce propiomelanocortin (POMC) and inhibit dopamine transmission [46,47,48]. Hence, it would be interesting to know the effect that EFV has on the hypothalamic–pituitary–adrenal axis.

A direct effect on the hypothalamic centers could be the cause of involuntary weight loss that leads to a lack of energy in the cells, which would increase food intake. Due to its wide distribution in the murine brain [28], and in accordance with our results observed in this work on the downregulation of the Tph2 gene in the hypothalamus (Figure 1A), it is likely that EFV causes alteration of other genes, such as those of the c-Jun N-terminal kinases (JNK1), which are widely expressed in the brain and responsible for regulating the amount of food that the organism ingests [49].

JNK1 blocks the hypothalamic-pituitary-thyroid axis, reducing energy expenditure and promoting obesity. JNK1 activation in hypothalamic agouti gene-related protein (AgRP) neurons induces an increase in intake and weight gain and impairs insulin and leptin signaling, JNK3 (MAPK10) deletion in the same neuronal population produces very similar effects [50], so it will be important to know the role of EFV on these genes and the hypothalamic–pituitary–thyroid axis for future studies. The regulation of body weight is multidimensional; it depends on metabolic, hormonal, and molecular factors (involving, among others, lipids, glucose, leptin, ghrelin, insulin, cortisol, thyroxine, growth hormone, serotonin, and some 5-HT receptors and their ligands). Although in this study we did not measure hormonal and biochemical profiles, some studies in PLWH showed that chronic administration of EFV causes changes in the levels of lipids, glucose, and cortisol, and those changes are related to age, where aging presents a high prevalence of metabolic diseases and weight gain [51,52,53,54]. In studies reported by Baza Caraciolo et al., in 2007, the lipid profile of HIV-positive patients without treatment was evaluated, where a marked decrease in HDL, ApoA1, and total cholesterol were significantly lower in HIV (+) patients independent of CD4 levels, finding significantly higher atherogenic risk regardless of the presence of other risk factors [55]. It must be taken into account that in the present study, the effect of the drug was evaluated without the presence of the virus. Therefore, it will be convenient to measure the metabolic profile after chronic administration of EFV to elucidate whether these biomolecules are altered and impact the increase in intake and loss of body weight. The reason underlying the dissociation between body weight and food intake observed in this work following EFV administration is largely unknown; hence, it is worth studying further.

## 4. Materials and Methods

### 4.1. Mice and Ethical Aspects

Healthy, 12- to 14-week-old adult CD1 male mice (*n* = 48), 24 per group (*n* = 6 per experiment) weighing 40–44 g from the local colony of the Instituto de Fisiología Celular, Universidad Nacional Autónoma de México (UNAM), kept under controlled conditions (12/12 h dark-light cycle; lights on 7:00–19:00 h, and a temperature of 22 °C) were used. Mice were housed in groups of six in filter-top cages (17.8 × 30.5 × 12.7 cm^3^) and provided food (LabDiet 5001 PMI^®^, Laboratory Rodent Diet, LabDiet, CA, USA) and water ad libitum.

Food consumption was measured using a digital scale (Lab-Tech^®^). The amount of food consumed per box (*n* = 6) was weighed daily at 2:00 p.m. The starting point was 400 g of food, the same day of starting EFV administration, and it was refilled each day with a standard amount (400 g) until 36 days.

This study was carried out according to the guidelines in the Guide for the Care and Use of Laboratory Animals established by Mexican Animal Welfare and Ethical Authorities (Norma Oficial Mexicana, 1999) [56]. The Ethics Animal Experiments Committee approved the protocol (MPM170-21) at the Instituto de Fisiología Celular, UNAM.

### 4.2. Pharmacological Treatment

Mice were randomly divided into two groups of twenty-four animals each. One group received distilled water (1.5 µL/kg) (control group), and the other group received EFV (SUSTIVA^®^ tablets, 600 mg, by Bristol-Myers Squibb Pharma, Montreal, QC, Canada) (10 mg/kg for 36 days) [25]. Distilled water and EFV were quickly and gently administered into the mouth through a metal cannula (mouse oral gavage of 3.0 mm diameter, 1.2 mm curve, and 55 mm length for the 18G syringe, Ketu Store) attached to a syringe. To avoid the toxic effects of the drug, the animals were weighed daily, and the drug volumes were adjusted to the animal weights. EFV was administered orally once a day for 36 days (for chronic administration) at a dose of 10 mg/kg, previously reported by Romão PR et al., 2011 [25]; and also based on the doses used for human therapy (dose: 600 mg daily) reported by Nadezda Apostolova et al., 2015, a review [57] and previous studies with animals [23].

Food intake was monitored daily to determine how EFV influences hunger/appetite. At the end of treatment, six animals from each group were anesthetized (pentobarbital, 40 mg/kg, i.p.) and quickly decapitated, and their brains were excised promptly from the skull. Regions of interest (brainstem, amygdala, and hypothalamus) were rapidly dissected. For the ELISA, dissected tissues were frozen on dry ice and stored at −80 °C until further analyses. For qPCR analysis of Tph2 gene expression, tissues were incubated in mRNA preservation with buffer solution (RNA Stabilization Reagent, Qiagen, Hilden, Germany) at −80 °C until analysis by qPCR using Taq Man probes (Applied Biosystems, Foster City, CA, USA).

### 4.3. RNA Preparation and Tph2 Expression Analysis by Quantitative Real-Time PCR

Frozen brain tissue was powdered, and total RNA was extracted using an RNeasy^®^ Mini Kit according to the manufacturer’s protocol (Qiagen, Valencia, CA, USA). qPCR analysis was performed using an Applied Biosystems 7300 Real-Time PCR system (Applied Biosystems, Foster City, CA, USA) in a 25 µL reaction mixture containing 100 pg of template RNA. PCR was performed using a One-Step RT-PCR Kit (Applied Biosystems, Foster City, CA, USA) for Tph2 (Mm00557717_m1) and the beta-actin (Actb) (Mm02619580_g1) TaqMan^®^ probes. The Actb gene was chosen as a reference gene. Real-time polymerase chain reactions were performed in triplicate. Reverse transcription was performed at 45 °C for 10 min, followed by reverse transcription inactivation at 95 °C for 10 min, and amplification for 40 cycles of 95 °C for 15 s. Primer annealing was performed at 60 °C for 45 s. The relative amount of mRNA in each sample was calculated by the comparative ΔCt method.

### 4.4. Brain 5-HT Measurements

To measure 5-HT levels in brain tissue, a commercial ELISA kit was used (MyBioSource, San Diego, CA, USA). Briefly, brain tissue was homogenized in PBS (pH 7.5) using a sonicator (Thermo Fisher Scientific, Kennesaw, GA, USA) (2 cycles of 30 s each) and centrifuged for 15 min at 5000 rpm and 4 °C. 5-HT quantification was performed in triplicate according to the manufacturer’s protocol, and the plate was read spectrophotometrically with an ELISA reader (Thermo Scientific TM, Kennesaw, GA, USA) at 450 nm. The 5-HT concentration in tissue was expressed in pg/mg tissue.

### 4.5. Behavioral Assays

#### 4.5.1. Forced Swim Test

At the end of treatment, six animals from each group were subjected to the forced swim test (FST). The FST was performed essentially by the method described by Yankelevitch et al. (2015) for mice. Plexiglas cylinders (50 cm high; 20 cm diameter) filled with water up to a height of 25 cm were used for their performance. The water was kept at 24 ± 1 °C. To follow the authors’ recommendation, although 6 min of swimming activity were videotaped, only the last 4 min were analyzed to prevent the high activity exhibited by the mice at the beginning of the test from contaminating the results of the test [58]. During the swimming sessions, immobility time was defined as the time the animal spends in the water without making any movements beyond those required to keep its head above the water. It should, however, be noted that unlike the conventional Porsolt test [59], swimming behavior, instead of being evaluated in a second session carried out 24 h after its initial exposure, was assessed in this test immediately after the animals were exposed to the water, but the behavior exhibited by the mice was also interpreted as passive coping or “hopeless” behavior.

#### 4.5.2. Elevated Plus Maze

At the end of treatment, six animals from each group were exposed to elevated plus-maze (EPM), which is a well-established animal model for testing anxiolytic-like and anxiogenic-like drugs. The maze used in this work was based on the design of Pellow et al. (1985) and was constructed as described by Pérez de la Mora et al. (2012) [60,61]. It consisted of two open arms (50 × 10 cm) and two enclosed arms (50 × 10 × 40 cm) with an open roof. The arms intersected at the central square (10 × 10 cm). The maze was elevated 50 cm from the floor by a pedestal joined to the central square. To prevent the mice from falling from the maze, wooden sledges (0.5 × 0.5 cm) were attached along the edges of the open arms. Mice were placed on the central square of the maze facing an open arm at the beginning of the test and were allowed to explore the maze for 5 min. The number of entries to the open arms (expressed as the percentage of the total number of arm entries) and the total time spent in these arms of the maze were taken as anxiety indices (the higher the index was, the lower the anxiety). The number of entries was counted when the four paws of the mice were placed in the respective arms. The maze was cleaned with detergent and dried before each trial. The illumination level at the central square of the maze was 100 l× during testing.

#### 4.5.3. Open-Field Test

At the end of treatment, six animals from each group were subjected to the open field test (OFT), which is used to analyze locomotion, anxiety, and stereotypical behaviors such as grooming and rearing in rodents [62]. For mice, the test area normally consists of a 42 × 42 cm polyvinyl chloride (PVC) box, and a camera is used to monitor movement into and around the central and peripheral areas of the box. Changes in locomotion can be indicative of altered neurological processes and may therefore reflect abnormal brain function. In addition, this test may be used to assess the general health and well-being of an animal. Animals that are not healthy tend to move less within the area. Mice that are stressed show less activity in the open field and increased stereotypical behavior [63,64]. Such behaviors include those that are repetitive, invariant, and seemingly without purpose. Mice that prefer to stay close to walls and travel more in the periphery can be described as showing thigmotaxis (movement towards a solid object), which is pronounced in mice showing signs of anxiety-like behavior. Mice with lower anxiety tend to spend more time in the central, open area of the box [65].

### 4.6. Statistical Analysis

The statistical analyses were performed using GraphPad Prism 6.0 statistical software (Inc., La Jolla, CA, USA). The distribution of the values of the studied parameters was tested for normality using the Shapiro–Wilk test. Tph2 expression, 5-HT levels, and behavioral data were analyzed by unpaired *t* tests with Welch’s correction to determine differences between the experimental group and the control group. Food intake was analyzed by unpaired *t* tests, and body weight was analyzed with a two-factor repeated-measures ANOVA followed by the Tukey post hoc test. All the data are expressed as the mean ± standard error (S.E.M.) of three independent triplicate assays. The statistical significance was set at *p* < 0.05.

## 5. Conclusions

In conclusion, our results showed that chronic administration of EFV to healthy mice led to Tph2 gene dysregulation within the brainstem, amygdala, and hypothalamus, as well as increased 5-HT levels in the amygdala, which resulted in more anxiety than behaviors related to depression. Further studies will be necessary to clarify the increase of 5-HT in the amygdala using another technique different from ELISA, such as HPLC, as well as understand the paradoxical decrease in body weight with the simultaneous increase in food consumption.

## Data Availability

The data are contained within the article.
